# Baseline insulin sensitivity affects response to high-amylose maize resistant starch in women: a randomized, controlled trial

**DOI:** 10.1186/s12986-016-0062-5

**Published:** 2016-01-13

**Authors:** Barbara A. Gower, Richard Bergman, Darko Stefanovski, Betty Darnell, Fernando Ovalle, Gordon Fisher, S. Katherine Sweatt, Holly S. Resuehr, Christine Pelkman

**Affiliations:** Department of Nutrition Sciences, University of Alabama at Birmingham (UAB), 616A Webb Building, 1675 University Blvd, Birmingham, AL 35294 USA; Cedars-Sinai Diabetes and Obesity Research Institute, Los Angeles, CA USA; New Bolton Center, School of Veterinary Medicine, University of Pennsylvania, Philadelphia, PA 19104 USA; Clinical Research Unit, UAB, Birmingham, USA; Department of Medicine, Division of Diabetes, Endocrinology, and Metabolism, UAB, Birmingham, USA; Department of Human Studies, UAB, Birmingham, USA; Department of Nutrition Sciences, UAB, Birmingham, USA; Ingredion, Incorporated, Bridgewater, NJ USA

**Keywords:** Minimal model, Intravenous glucose tolerance test

## Abstract

**Background:**

Resistant starch (RS) is a type of dietary fiber that can improve glucose metabolism, but its effects may be modulated by sex or baseline insulin sensitivity. This study was designed to examine the effect of high-amylose maize resistant starch (HAM-RS2) on insulin sensitivity (S_I_) in women, and to determine if S_I_ status affects the response to RS.

**Methods:**

This was a randomized, placebo-controlled, double-blind, cross-over study. Participants were 40 healthy, non-diabetic women aged 22–67 years in the normal-weight to obese BMI range (20.6–47.4 kg/m^2^). Two doses of HAM-RS2 were tested, 15 and 30 g per day, administered in the form of cookies. Participants were randomized to the order in which they received the experimental and placebo product. Each arm was 4 weeks, with a 4-week wash-out period in between. S_I_ was assessed at the end of each 4-week arm of product consumption by frequently-sampled, insulin-modified, intravenous glucose tolerance test and minimal modeling. Participants were categorized as being insulin resistant (IR; S_I_ < 7.8) or insulin sensitive (IS; S_I_ ≥ 7.8) based on Gaussian analysis. The effect of treatment arm on S_I_ was examined by mixed-model analysis within IR and IS sub-groups, using all available data. In addition, S_I_ was examined by ANOVA among just those women who completed all three arms of the study with valid S_I_ results.

**Results:**

Among IR participants, S_I_ was on average ~16 % higher after the 30 g arm when compared to the control arm by mixed-model analysis (*n* = 40, *P* < 0.05), and tended to be 23 % higher by ANOVA among women who completed all arms (*n* = 23, *P* = 0.06). HAM-RS2 did not affect S_I_ in IS women.

**Conclusion:**

Consumption of HAM-RS2 at 30 g/day in the form of a snack food item was associated with improved insulin sensitivity in women with insulin resistance.

**Clinical trials registry number:**

NCT0152806.

## Background

Resistant starch (RS) is a type of dietary fiber that has beneficial effects on insulin sensitivity and gastrointestinal health in humans [1]. By definition, RS is a type of carbohydrate that is incompletely digested by human pancreatic amylases. As a result, the starch molecules are cleaved in the colon by bacterial enzymes and subsequently metabolized as fuel by the gut microbiota. Four types of RS have been identified. Type 2, which is found in some starchy foods such as green bananas, potatoes, and high-amylose corn, has been investigated most extensively for its effects on human health.

It is thought that the insulin-sensitizing effects of RS are due in part to the short-chain fatty acids that result from bacterial metabolism of the carbohydrate molecules [2]. Administration of exogenous short-chain fatty acids results in suppression of circulating free fatty acids by limiting lipolysis [3]. Because elevated fatty acids are associated with both suppression of insulin-stimulated glucose uptake at skeletal muscle [4] and inhibition of insulin suppression of glycogenolysis at the liver [5], their reduction may improve insulin sensitivity. It is also possible that RS increases adiponectin, an adipocyte-derived hormone with insulin sensitizing properties. In mice, RS increases adipose tissue adiponectin concentrations [6], and in humans, greater intake of cereal fiber is associated with greater circulating adiponectin [7, 8].

Clinical studies documenting effects of RS on insulin sensitivity have generally yielded positive results. Acute consumption of 60 g RS over 24 h resulted in 69 % higher insulin sensitivity by mixed-meal tolerance test in a group of 10 healthy men and women [9]. Consumption of type 2 RS for 4 weeks at a dose of 30 g/day was associated with a 33 % increase in insulin sensitivity as assessed with mixed-meal tolerance test, and a 14 % increase as assessed by euglycemic clamp in a group of 10 healthy men and women [10]. Consumption of 40 g/d RS led to a 16 % increase in insulin sensitivity by euglycemic clamp in a group of 20 insulin resistant men and women [11]. Consumption of both 15 and 30 g/d RS for 4 weeks was associated with increased insulin sensitivity (48 and 53 %, increase in mean values, respectively) as assessed with intravenous glucose tolerance test in a group of 11 overweight/obese men [12]. However, in the same study, no effect was observed in a group of 22 women, leading to a significant treatment-by-sex effect (*P* < 0.05). Although the explanation for this sex difference was not known, it was speculated that failure to control for menstrual cycle phase, or higher insulin sensitivity in women vs men [13, 14], may have played a role.

This study was designed to examine the effect of high-amylose maize resistant starch (HAM-RS2) administered as a snack food on insulin sensitivity (S_I_) in women, controlling for menstrual cycle phase, and to determine if S_I_ status (high vs low) affects the response to RS.

## Methods

Participants were healthy, sedentary women. Exclusion criteria included type 1 or type 2 diabetes, polycystic ovary syndrome, disorders of glucose or lipid metabolism, hypertension, use of medication that could affect glucose metabolism (including non-approved oral contraceptives and postmenopausal hormone replacement therapy), use of tobacco, alcohol consumption in excess of 400 grams per week, engagement in more than 2 h intentional moderate or light exercise per week, and a medical history that counter-indicated inclusion in the study. Women were categorized as premenopausal if they experienced regular menstrual cycles, and postmenopausal if they were over age 50 and had not had a cycle in the past 12 months. One woman aged 53 reported irregular menstrual cycles; she was categorized as peri-menopausal and coded with the premenopausal group for statistical analyses. Premenopausal women were permitted to use tri-phasic, low-dose oral contraceptives. Because one goal of the study was to determine if baseline insulin sensitivity status affected the response to RS, an effort was made to recruit African-American women, who are more insulin resistant than Caucasian women, even when obesity status and fat distribution are accounted for [15, 16]. Thus, including both African-American and Caucasian women would increase the likelihood of having a wide range of values for insulin sensitivity. Participants were informed of the experimental design, and oral and written consent were obtained. The study was approved by the Institutional Review Board for Human Use at the University of Alabama at Birmingham (UAB).

The study was conducted using a randomized, placebo-controlled, double-blind cross-over design. Two doses of resistant starch were tested, 15 and 30 g per day, administered in the form of crackers, and vanilla- and lemon-flavored cookies. Within the first two months of the trial, subjects indicated a dislike for the crackers, and the study continued with cookies only. The resistant starch was a type 2, granular, form from high-amylose maize (HAM-RS2). A control snack was formulated with a highly digestible waxy corn starch to match the amount of digestible starch provided by the HAM-RS2 in the 15 g snack (~11.6 g/d). The cookies were tested to verify macronutrient, fiber, and RS content (Table 1). Each arm was 4 weeks, with a 4-week wash-out period between. Test products were administered in random order. For all arms, participants were asked to consume two servings of test product per day (28–47 g per serving, depending upon the snack type and starch dose). Participants reported to the clinic weekly where they were weighed, met with the dietitian, and picked up product. If they missed a pickup, they were queried regarding their product use during the interim. If participants missed more than 2 days of product use, they either started over with that particular arm, or were removed from the study. Habitual diet was monitored via 4-day food records obtained at baseline and during each of the three test phases, analyzed with Nutrition Data Systems for Research (NDSR 2012). For premenopausal women, testing was conducted in the first 10 days of the follicular phase of the menstrual cycle. Anthropometrics were collected at baseline and after each arm. Height and weight were assessed using an electronic scale and a wall-mounted stadiometer. Waist circumference was measured around the narrowest portion of the torso with a flexible tape.

**Table 1:** Ingredient list and composition of test cookies^a^

	Control	15 g RS	30 g RS
Ingredients (g/100 g)			
All-purpose flour	24.8	16.5	0.8
Wheat gluten	7.9	4.7	3.9
Sunflower oil	8.2	5.9	4.3
Salt	0.09	0.08	0.08
Sugar	13.3	13.4	12.9
Lemon extract	2.1	2.1	2.1
Whole eggs	10.3	10.5	10.7
Baking powder	0.4	0.4	0.4
Water	9.3	11	14.8
Dry milk	7.4	7.4	7.6
Waxy corn starch^b^	16.1	0	0
HAM-RS2^c^	0	27.7	42.2
Composition (post-baking)			
Portion size (g)	28.4	36.7	46.3
Energy (kcal)^d^	126.9	129.6	138.2
Carbohydrate (g)^d^	18.5	19.2	20.8
Protein (g)^d^	4.7	4.6	5.0
Fat (g)^d^	3.8	3.8	3.9
Dietary fiber (g)^d^	0.5	7.8	14.8
Resistant starch (g)^e^	3.18	11.35	19.05

^a^ Cookies produced by International Food Network, Ithaca, NY. Data shown are for lemon-flavored cookies. Vanilla-flavored cookies were of similar formulation and composition (<3 % variation in composition between types of cookies; data not shown). Two servings of cookies were consumed per day

^b^AMIOCA® corn starch, Ingredion Incorporated, Bridgewater, NJ

^c^ HI-MAIZE® 260 resistant starch, Ingredion Incorporated, Bridgewater, NJ

^d^ Composite analyses performed by Medallion Labs, Minneapolis, MN; Dietary fiber determined using AOAC method 991.43

^e^ Resistant starch estimated using a modified Englyst RS assay

Insulin sensitivity was assessed by insulin-modified, frequently-sampled intravenous glucose tolerance test (IVGTT) and Minimal Model analyses [17–19]. Testing was conducted in the Clinical Research Unit of UAB’s Center for Clinical and Translational Science (CCTS) after an overnight fast. Prior to testing, flexible intravenous catheters were placed in the antecubital spaces of both arms. Three, 2.0 ml blood samples were taken over a 20-min period for determination of basal glucose and insulin (the average of the values was used for basal “fasting” concentrations). At time “0”, glucose (50 % dextrose; 300 mg/kg) was administered intravenously over 2 min. Insulin (0.02 U/kg, Humulin, Eli Lilly and Co., Indianapolis) was infused from 20 to 25 min post glucose injection. Blood samples (2.0 ml) were then collected at the following times (min) relative to glucose administration: 2, 3, 4, 5, 6, 8, 10, 12, 15, 19, 20, 21, 22, 24, 26, 28, 30, 35, 40, 45, 50, 55, 60, 70, 80, 100, 120, 140, 180, 210, 240.

Sera were stored at -85 °C until analyzed. Glucose and insulin values were entered into the MINMOD computer program (Millennium version, © Richard N. Bergman [19]) for determination of the insulin sensitivity index (S_I_). The acute insulin response to glucose (AIRg) was calculated by the software as the incremental insulin area-under-the-curve from minutes 0–10 following glucose injection using the trapezoidal method. Disposition Index (DI) was calculated as S_I_ x AIRg, a composite measure of insulin secretion and insulin sensitivity. Glucose effectiveness (S_G_) also was generated by the model; S_G_ describes the extent to which glucose uptake is stimulated, and glucose production inhibited, by glucose itself, independent of a dynamic change in insulin.

Laboratory analyses were conducted in the Core Laboratory of the CRU, Nutrition Obesity Research Center, and Diabetes Research Center. Glucose, total cholesterol, HDL-cholesterol, and triglycerides were measured using a SIRRUS analyzer (Stanbio Laboratory, Boerne, TX). Insulin was assayed by immunofluorescence on a TOSOH AIA-II analyzer (TOSOH Corp., South San Francisco, CA); intra-assay CV of 1.5 % and inter-assay CV of 4.4 %. Adiponectin was assessed by radioimmunoassay (Linco-Millipore; Billerica, MA); inter-assay CV was 9.98 %; intra-assay CV was 4.70 %; and sensitivity (90 % bound) was 1.0 μg/ml.

**Fig. 1:**
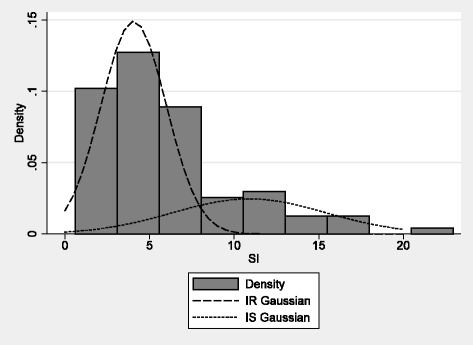
Gaussian distributions of insulin sensitivity values. Two groups [(insulin resistant (IR) and insulin sensitive (IS)] were identified based on the cut-point of 7.8

IR women were identified using histogram analysis of S_I_ values from all valid tests, and univariate normal mixture decomposition analysis. Two subgroups were noted, with the majority of observations belonging to a distribution with a mean of 4.1 and a SD of 1.9. A cut-point for IR was established at 7.8 based on the estimated SD for this group, which was two standard deviations from the observed mean (upper limit on the 95 % confidence interval, Fig. 1). Thus, for subsequent analyses, participants were categorized as being either IR (S_I_ < 7.8) or insulin sensitive (IS; S_I_ ≥ 7.8). Eight women in the group who completed all three phases of the study had at least one S_I_ measure ≥7.8.

Data were analyzed by mixed-effects modeling within each subgroup (IR/IS), with waist circumference and completer status as covariates, subject ID as a group variable, and both test doses of RS contrasted against the placebo treatment. The mixed model considers all data, and thus allowed for inclusion of data from women who completed only one or two arms of the study. To confirm that the results obtained with the mixed model were valid within women who completed all three arms of the study, results also were analyzed by repeated-measures ANOVA and Student-Newman-Keuls pairwise comparisons within only those women who had valid S_I_ tests for all three arms (*n* = 23). Descriptive information was compared by ANOVA between IR and IS sub-groups using the Dose = 0 data. Ancillary, exploratory, analyses were conducted within sub-groups based on menopausal status and race/ethnicity. Due to small sample sizes in these sub-groups, paired *t*-test was used to explore differences in S_I_ among the three RS doses. For all analyses, *P* < 0.05 was considered significant.

## Results

**Fig. 2:**
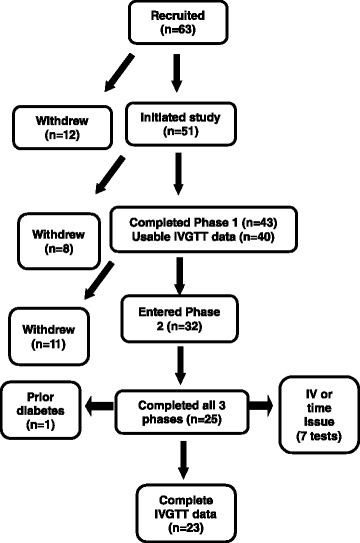
Consort diagram depicting participant numbers throughout recruitment and testing

Fifty-one women entered the study (Fig. 2), and 43 completed the first phase of the study, 40 of whom had a usable S_I_ result. For mixed-model analysis, data from these 40 women were used. The mean age of this sample at baseline was 48.3 ± 12.6 years; mean BMI was 29.8 ± 6.7 kg/m^2^. Eighteen women were pre- or peri-menopausal; 22 were postmenopausal. The ethnic composition was 24 non-Hispanic Caucasian, 14 African-American, and 2 Hispanic. Twelve women had at least one S_I_ measure >7.8; all were Caucasian. Twenty-five women completed all three phases of the study; of these, 24 had usable S_I_ results. Seven insulin sensitivity tests were not available for issues relating to iv access or scheduling. Reasons for discontinuing the study included difficulty with the time commitment, transportation problems, and unwillingness to consume the snacks as directed. Data from one woman were excluded due to IVGTT results indicating undiagnosed diabetes. For ANOVA, data from the 23 non-diabetic women who completed all three phases of the study and had usable S_I_ results were used. The mean age of this sample at baseline was 52.4 ± 12.0 year. Seven women were pre- or peri-menopausal; 14 were postmenopausal. The ethnic composition was 17 non-Hispanic Caucasian and 6 African-American. All IS women were Caucasian, and all African-American women were in the IR group. The IR group contained 4 pre-and 10 post-menopausal women. The IS group contained 3 pre- and 6 post-menopausal women.

Thirty-one women completed food records during at least one arm of the study, for a total of 68 records. Analysis of diet data indicated that, on average, habitual diet did not change across the three arms of the study, and was not different between the IR and IS sub-groups for any treatment arm. Combined average intake of total energy was 1665 ± 496 kcal/day; %energy from carbohydrates, protein, and fat was 45.7 ± 7.1, 16.4 ± 3.8, and 35.9 ± 5.0, respectively. Intake of dietary fiber (g/day; not including the experimental product) was not different across treatment arms. However, daily intake of total dietary fiber was significantly and consistently lower in the IR vs the IS women across the entire course of the study: Dose = 0 g/day (13.9 ± 4.1 vs 18.0 ± 4.4, *P* < 0.05); Dose = 15 g/day (14.0 ± 6.6 vs 21.3 ± 1.0, *P* < 0.05); and Dose = 30 g/day (13.4 ± 3.7 vs 19.0 ± 5.9, *P* < 0.01).

**Fig. 3:**
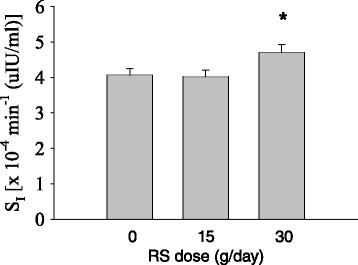
Insulin sensitivity (S_I_; mean ± SEM) by dose at the end of each 4-week arm within insulin resistant women (*n* = 28). *Dose = 30 g/day differed from Dose = 0 g/day (*P* < 0.05) by mixed-model analysis. Means ± SEM were 0: 4.07 ± 0.18; 15: 4.02 ± 0.19; 30: 4.70 ± 0.23

When considering the entire group of 40 women who completed at least one arm of the study, mixed model analysis among IR participants (*n* = 28) revealed a significant impact of the 30 g dose of RS (*P* < 0.05; Table 2, Fig. 3). Predicted values (Mean ± SEM) by dose were 0: 4.07 ± 0.18; 15: 4.02 ± 0.19; 30: 4.70 ± 0.23. No effect of either RS dose was observed among IS participants (*n* = 12). No carryover effects were detected.

**Table 2:** Mixed-effects model for the dependent variable S_I_^a^ within all IR women in the study (S_I_ < 7.8; *n* = 28)

	Coefficient	SEM	*P*	95 % CI
15 g/d	0.31	0.36	0.38	-0.39–1.01
30 g/d	0.87	0.38	0.02	0.12–1.62
Completer status^b^	0.65	0.51	0.20	-0.34–1.65
Waist circumference	-0.07	0.02	0.00	-0.11–0.03
Constant	10.20	1.86	0.00	6.56–13.83

*S*_*I*_ insulin sensitivity

^a^S_I_ was obtained after 4 weeks of treatment with 0, 15 g, or 30 g RS/day. Subject ID was included as a group variable

^b^Participants were coded “0” if they completed one or two arms, or “1” if they completed all three arms of the study

Data from those women who completed all three arms of the study are shown in Table 3, by IR/IS status, at the end of each study arm. On average, IR women had higher concentrations of fasting glucose and insulin than IS women (at dose = 0). Waist circumference at dose = 0 (*n* = 18) averaged 99.2 ± 12.2 in the IR sub-group, and 90.7 ± 12.3 in the IS sub-group (*P* = 0.184). Body weight at dose = 0 was higher in the IR group (86.8 ± 16.6 kg) than in the IS group (67.0 ± 8.0 kg) (*P* < 0.01). BMI at dose = 0 was higher in the IR group (32.9 ± 7.3 kg/m^2^) than in the IS group (25.6 ± 3.4 kg/m^2^) (*P* < 0.05).

**Table 3:** Biochemical and metabolic measures of the 23 women who completed all arms of the study by insulin sensitivity sub-group (IR/IS) after 4 weeks of exposure to the indicated dose of RS. Mean (SD)

	IR (*n* = 14)			IS (*n* = 9)		
	RS dose (g/day)			RS dose (g/day)		
	0	15	30	0	15	30
Glucose (mg/dL)*	100.0 (12.3)	98.3 (9.4)	98.2 (13.0)	87.9 (4.5)	86.9 (6.6)	91.6 (8.5)
Insulin (μIU/ml)**	10.3 (4.6)	9.8 (4.6)	9.6 (6.9)	4.0 (1.8)	4.7 (2.0)	4.6 (1.4)
S_I_ [(×10^-4^ min^-1^ (μIU/ml)]***	3.80 (1.54)	3.61 (1.60)	4.68 (1.71)	10.59 (5.77)	8.73 (3.61)	8.56 (2.95)
AIRg (μIU/mlx10 min)	510.6 (378.9)	438.1 (342.8)	582.1 (524.4)	380.0 (339.4)	344.1 (257.3)	403.3 (308.6)
DI	2000.2 (2221.0)	1444.7 (1197.6)	2680.8 (2913.2)	3342.9 (2507.4)	2746.7 (2132.2)	3470.9 (2992.3)
S_G_ (min^-1^)	0.020 (0.015)	0.015 (0.008)	0.021 (0.013)	0.022 (0.007)	0.027 (0.014)	0.023 (0.011)
Total Chol. (mg/dL)	187.6 (38.3)	181.2 (24.4)	190.4 (37.4)	179.6 (32.2)	182.4 (34.1)	190.8 (41.9)
HDL-C (mg/dL)	59.0 (18.0)	58.8 (9.6)	56.9 (14.3)	61.9 (8.1)	62.7 (8.9)	62.9 (12.9)
TG (mg/dL)	111.7 (47.9)	111.8 (50.8)	118.2 (59.1)	83.7 (19.6)	79.1 (27.0)	96.1 (31.1)
Adiponectin (μg/mL)	11.4 (5.7)	11.4 (4.9)	11.5 (5.4)	16.2 (4.6)	16.1 (4.3)	17.7 (6.8)

*P < 0.05, **P < 0.001 for IR vs IS at dose = 0 g/day

***P < 0.05 for dose effect within the IR sub-group. The 30 g/day dose differed from the 15 g/day dose (*P* < 0.05) and tended to differ from the 0 g/day dose (*P* = 0.068)

*IR* insulin resistant [S_I_ value <7.8 ×10^-4^ min^-1^ / (μIU/ml)]

*IS* insulin sensitive [S_I_ value ≥7.8 ×10^-4^ min^-1^ / (μIU/ml)]

*S*_*I*_ insulin sensitivity by minimal model

*AIRg* acute insulin response to glucose

*DI* disposition index (dimensionless)

*S*_*G*_ glucose effectiveness

*TG* triglycerides

ANOVA within the IR sub-group indicated a significant main effect (*P* < 0.05) of “Dose” on S_I_. Post-hoc comparison among doses indicated that the 30 g/day dose differed from the 15 g/day dose (*P* < 0.05); the comparison of the 30 and 0 g doses approached significance (*P* = 0.068). No dose effect was observed within the IS sub-group. Further analyses indicated that the significant effect of RS was confined to the postmenopausal women (34 % higher after 30 g RS vs 0 g RS, *P* < 0.05). When secondary outcomes were examined within the IR sub-group, the Disposition Index (DI; *P* < 0.05) and AIRg (*p* = 0.056) were higher with the 30 g/day dose compared to the 15 g/day dose. No differences were observed within the IS sub-group.

## Discussion

The primary finding from this study was that 30 g/d of RS in the form of a snack food led to greater insulin sensitivity among IR women, a group that included all of the African-American women in the study. These results extend observations from clinical studies involving samples of healthy men and women combined, men and women with metabolic syndrome, and overweight/obese men, among whom beneficial effects of RS on insulin sensitivity have been detected. Importantly, results from this study may explain the significant treatment-by-sex interaction observed previously [12], where RS treatment increased insulin sensitivity in obese men but not women. Based on the present results, we tentatively conclude that RS is most effective in insulin resistant populations.

One of the major findings of the study was that an effect of baseline insulin sensitivity was detected, such that RS treatment improved insulin sensitivity among IR but not IS women. The IR group included all of the African-American women enrolled in the study. In contrast, the IS group was exclusively Caucasian. African-Americans are at disproportionate risk for type 2 diabetes, and we have previously reported that S_I_ is lower in African-Americans when compared to Caucasians [15, 16]. We also observed that the effect of RS on S_I_ was significant only within the postmenopausal women in the IR sub-group. In these women, a significant (*P* < 0.05) 34 % increase in S_I_ occurred between the 0 and 30 g/day doses. After menopause, women experience increases in risk for several metabolic diseases including type 2 diabetes and cardiovascular disease [20, 21]. Although further studies with larger samples are needed before conclusions can be drawn, results of this study suggest that RS may be particularly effective at improving insulin sensitivity in African-Americans and post-menopausal women, groups that are at elevated risk for chronic metabolic disease.

We noted that the IR sub-group, on average, consumed 23–33 % less dietary fiber than did the IS sub-group. Further, in all women combined, dietary fiber intake was positively associated with insulin sensitivity (*r* = 0.28, *P* < 0.05) in a simple correlation analysis (data not shown). Although these results must be treated with caution because compliance with food record submission was incomplete, it is possible that lower insulin sensitivity in the IR sub-group was due at least in part to habitually lower fiber consumption. It is also possible that the lack of effect of the RS treatment on insulin sensitivity in the IS sub-group was due to their greater habitual consumption of dietary fiber.

Although two doses of RS (15 and 30 g/day) were tested in this study, the effect of RS was significant only with the 30 g/day dose. In contrast, within abdominally obese men, consumption of both 15 and 30 g/d RS for 4 weeks resulted in higher mean S_I_ [12]. The IR women in this study had a waist circumference of ~99 cm on average, a value that corresponds to a visceral fat area of >130 cm^2^, the threshold for perturbations in glucose-insulin homeostasis [22]. Nonetheless, these women were relatively healthy. It is possible that a cohort of women with greater metabolic dysfunction would have responded to the lower dose of RS. It is also possible that women are less responsive to RS, and therefore require a higher dose.

In this study, IR women exhibited greater insulin sensitivity after the 30 g/d treatment compared to placebo. This insulin sensitivity was at the “whole body” level; i.e., the Minimal Model S_I_ value includes both insulin-stimulated glucose uptake at skeletal muscle and insulin inhibition of hepatic glucose production. Thus, we cannot determine whether RS treatment affected skeletal muscle, liver, or both. A previous study that used a tracer-based euglycemic clamp indicated that RS affected skeletal muscle and adipose tissue but not hepatic insulin sensitivity in insulin resistant participants [3]. Nonetheless, several studies have reported lower fasting glucose and/or insulin, markers of hepatic insulin sensitivity, following treatment with RS [3, 23]. Further, the effect of RS on whole-body insulin sensitivity is relatively large (e.g., 33–69 % observed with meal tolerance test or intravenous glucose tolerance test [9, 10, 12]) in contrast to the 14–16 % improvement observed with the muscle-specific euglycemic clamp [10, 11]. Studies are needed to more extensively characterize the site of RS action, and determine whether the site of action differs with the metabolic phenotype of the participants.

Strengths of this study were the double-blind, randomized, placebo-controlled cross-over design; inclusion of African-American women, a group at disproportionate risk for type 2 diabetes; testing during only the follicular phase of the menstrual cycle; administration of two doses of RS; and administration of the RS as a snack food, which increases the translational potential of the results. The main limitation was that many of the women did not complete all three arms of the study. This was a demanding study, requiring women to commit to almost 6 months of involvement, with at least 12 weeks of product consumption, and three intravenous glucose tolerance tests. In addition, some women tired of consuming two servings of cookies each day; a greater variety of snack choices may have increased adherence. The small sample size made interpretation of sub-group analyses difficult; a larger sample size is needed to more closely examine the response of sub-groups based on insulin sensitivity status, menopausal status, and ethnicity/race.

## Conclusions

In conclusion, among IR women, consumption of RS at a dose of 30 g/d in the form of a snack food item was associated with improved insulin sensitivity. RS may be an appropriate dietary ingredient to improve insulin sensitivity in women, particularly those at elevated risk for type 2 diabetes, such as African-American and post-menopausal women.
