# Erratum to: Baseline insulin sensitivity affects response to high-amylose maize resistant starch in women: a randomized, controlled trial

**DOI:** 10.1186/s12986-016-0065-2

**Published:** 2016-02-01

**Authors:** Barbara A. Gower, Richard Bergman, Darko Stefanovski, Betty Darnell, Fernando Ovalle, Gordon Fisher, S. Katherine Sweatt, Holly S. Resuehr, Christine Pelkman

**Affiliations:** Department of Nutrition Sciences, University of Alabama at Birmingham (UAB), 616A Webb Building, 1675 University Blvd, Birmingham, AL 35294 USA; Cedars-Sinai Diabetes and Obesity Research Institute, Los Angeles, CA USA; New Bolton Center, School of Veterinary Medicine, University of Pennsylvania, Philadelphia, PA 19104 USA; Clinical Research Unit, UAB, Birmingham, USA; Department of Medicine, Division of Diabetes, Endocrinology, and Metabolism, UAB, Birmingham, USA; Department of Human Studies, UAB, Birmingham, USA; Department of Nutrition Sciences, UAB, Birmingham, USA; Ingredion, Incorporated, Bridgewater, NJ USA

After corrections were submitted for this article [1], the authors realised the clinical trials registry number which they provided was incorrect and was therefore published incorrectly in the article [1] as NCT0152806. The correct clinical trials registry number is: NCT01521806. This has also been updated in the original article [1].
